# Mr. Purton, on the Treatment of Burns and Scalds

**Published:** 1808-12

**Authors:** T. Purton

**Affiliations:** Member of the Royal College of Surgeons in London. Alcester


### *To the Editors of the Medical and Physical Journal.*

Gentlemen,

I Have long since intended giving you my sentiments on a subject which has taken up many pages of your Journal, namely, the comparative good effects of the cooling with the the stimulating plan of cure in Burns and Scalds. It is certainly the duty of every medical man, to bring forward his practice on this particular point, as the only probable means of settling the matter in dispute. I coincide with several of your correspondents, that if both plans were occasionally made use of in certain cases, it might be attended with some advantage; for instance, where the scalding matter has diffused itself over the eyes, there the cooling means might be preferable, although I have had one case where the whole contents of a saucepan of boiling butter were accidentally dashed in a young woman's face, but being fortunately upon the spot, I immediately applied the ol. terebinth. by means of a brush of feathers, keeping the eye-lids closed for about ten minutes, or a quarter of an hour. I afterwards directed a folded linen rag to be moistened with the same, and kept upon the part until the burning sensation had subsided; the result was favourable, there was only a small vesication upon the neck, which was overlooked, and therefore had not had the advantage of the lotion. Again, as cold water is almost always at hand, plunging the part, if it should be one of the extremities, into it, at least until other means could be procured, is certainly a consideration which ought not to be undervalued.

In the first years of my practice, I pursued the cooling method, but for some considerable time I have made use of the terebinthinate application, and with that pleasing and happy success, that I shall not be readily induced to alter it. In my opinion, which is founded upon the successful issue of a number of cases, the stimulating plan has considerably the advantage, in as much as *permanent* ease is more readily procured than by the application of cold, and the secretion of pus also takes place *sooner*.

These, Gentlemen, are advantages not of little moment; and I do assure you, that since I have had recourse to Dr. Kentish's method, I have not been under one single instance of embarrasment, but on the contrary, have been uniformly charmed with the *immediate* ease produced; and several of my patients, who have had opportunities of trying both methods, voluntarily give it in favour of the new one, and that in terms of the highest eulogy. It is surely unnecessary to add more; what I have stated are facts. Cessation from pain is *almost instantly* produced by the terebinthinate application; but by the cooling treatment, it is necessary *often* to have a *constant renewal* of cold, for hours, before similar effects take place. I have been in the habit of using the ol. terebinth. cold, mixed with ol. lini in the proportion proportion of two parts of the former to one of the latter; and from my experience, applying it cold, has been attended with more beneficial effects than hot, although I can readily perceive that in bad cases of burns from metallic substances, where deep eschars are formed, that heating the oil might be attended with more advantage, but I have not yet had opportunities enough in my own practice to establish the fact.

I am, &c.

T. PURTON,
Member of the Royal College of Surgeons in London.

